# Use of low-value cancer care in practices acquired by private equity firms

**DOI:** 10.1093/jnci/djag093

**Published:** 2026-03-30

**Authors:** Aaron P Mitchell, Miranda B Lam, Caroline Carlin, Kevin Tyan, Maryam Mooghali, Ziad Zakaria, Reshma Ramachandran, Jeah Jung

**Affiliations:** Department of Epidemiology and Biostatistics, Memorial Sloan Kettering Cancer Center, New York, NY, United States; Department of Radiation Oncology, Brigham and Women’s Hospital, Boston, MA, United States; Department of Family Medicine and Community Health, University of Minnesota, Minneapolis, MN, United States; Department of Medicine, Harvard Medical School, Boston, MA, United States; Department of Internal Medicine, Yale School of Medicine, New Haven, CT, United States; Yale Collaboration for Regulatory Rigor, Integrity, and Transparency, Department of Medicine, Yale School of Medicine, New Haven, CT, United States; Department of Epidemiology and Biostatistics, Memorial Sloan Kettering Cancer Center, New York, NY, United States; Department of Internal Medicine, Yale School of Medicine, New Haven, CT, United States; Yale Collaboration for Regulatory Rigor, Integrity, and Transparency, Department of Medicine, Yale School of Medicine, New Haven, CT, United States; Department of Health Administration and Policy, College of Public Health, George Mason University, Fairfax, VA, United States

## Abstract

Private equity acquisition of medical practices has been associated with increased prices and use of lucrative treatments. Using Medicare data from 2015 to 2021, we compared the prevalence of low-value cancer treatments between private equity–owned and non–private equity–owned practices. The 5 low-value treatments were (1) granulocyte colony-stimulating factors for low-risk chemotherapy, (2) denosumab for castration-sensitive prostate cancer, (3) nab-paclitaxel for breast or lung cancer, (4) the addition of bevacizumab for ovarian cancer, and (5) biologic agents with biosimilar alternatives. The primary outcome was receipt of a low-value treatment. Comparing patients diagnosed when their oncologist was vs was not practicing at a private equity–owned address, adjusted prevalence differences of low-value treatments were −2.8 percentage points (95% confidence interval [CI] = −5.5 to −0.2) for granulocyte colony-stimulating factors, +3.7 percentage points (95% CI = −7.3 to 14.7) for denosumab, −0.7 percentage points (95% CI = −3.9 to 1.5) for nab-paclitaxel, −6.5 percentage points (95% CI = −12.5 to −0.4) for bevacizumab, and −2.9 percentage points (95% CI = −6.0 to 0.2) for biologics. Overall, private equity ownership was not associated with increased prevalence of low-value cancer treatments.

Private equity firms commonly invest in US health-care practices, seeking to generate financial returns by consolidating care delivery,^1^ reducing overhead costs, and increasing revenue.^2^ Although private equity acquisition may generate operational efficiencies and provide capital for struggling practices, it has been associated with staffing reductions, increased prices,^3-8^ and the selling of real estate assets,^9^ which may negatively affect care quality.^10^

The proportion of health-care practices owned by private equity firms has increased for many specialties.^3^ Oncology has recently become a target of such investment, with approximately 10% of medical oncologists and 15% of radiation oncologists practicing in private equity–owned clinics by 2022.^11^ Private equity ownership in oncology has grown rapidly, with limited evidence on utilization and quality.

Cancer treatments that are clinically unnecessary or have lower-cost alternatives result in avoidable costs and worse patient outcomes (through both drug toxicity and financial toxicity) but may still be lucrative to the physician. Physicians may be reimbursed a higher margin, even for low-value services, creating a potential financial incentive. Because private equity firms seek short-term financial returns, they may increase use of lucrative but low-value services. The goal of this study was to assess the association between private equity acquisition and use of low-value cancer treatments.

We examined 5 low-value cancer treatments based on prior literature or recommendations by professional societies: 2 nonrecommended treatments and 3 high-cost treatments with lower-cost alternatives (Table S1).^12^^,^^13^ The nonrecommended treatments were (1) granulocyte colony-stimulating factors (G-CSFs) for patients receiving low-risk chemotherapy and (2) denosumab for castration-sensitive prostate cancer. The high-cost treatments were (1) nab-paclitaxel instead of paclitaxel among patients with breast or lung cancer, (2) the addition of bevacizumab to carboplatin plus paclitaxel for ovarian cancer, and (3) use of an originator biologic instead of a biosimilar alternative (Table S2).

We used 100% traditional Medicare claims and Medicare Advantage national encounter data from 2015 through 2021. Following prior work,^14^ we used Medicare Advantage encounter data from contracts with complete records.

We included Medicare beneficiaries with a new cancer diagnosis from January 1, 2016, to June 30, 2020, and no cancer diagnosis or treatment claim for at least a 1-year washout period (Supplementary Methods A1). We required continuous Medicare parts A, B, and D (traditional Medicare patients) or parts C and D (Medicare Advantage patients) coverage during washout and follow-up. The index date was the first cancer diagnosis after washout. We identified cancer sites based on a hierarchical algorithm validated against Surveillance, Epidemiology, and End Results Program data.^15^ We then identified subcohorts corresponding to each low-value treatment (Supplementary Methods A2).

Within each subcohort, the outcome was receipt of the low-value cancer treatment for which patients in that subcohort were at risk. Each outcome was binary and observed only once for each patient. Patients could be included in multiple subcohorts if they met the requirements of each (eg, a patient might be assessed for both G-CSF use (G-CSF subcohort) and biologic vs biosimilar G-CSF use (biosimilar cohort).

The main explanatory variable was treatment at a private equity–owned practice (Supplementary Methods A3). First, patients were assigned an oncologist (National Provider Identifier designation) based on the plurality of evaluation and management claims in proximity to the index date, restricted to physicians with a medical oncologic specialty.^13^ Physicians’ historical practice addresses were then mapped to those identified as private equity acquired (data from Pitchbook and other public sources, including press releases).^11^ Using dates of both private equity acquisition and practice address changes, we identified (1) physicians who had ever practiced at a private equity–acquired address and (2) the time intervals during which they were and were not at a private equity–acquired address.

Patient-level covariates included age, sex, race, Medicare/Medicaid dual eligibility, insurance type (Medicare Advantage or traditional Medicare), cancer metastasis, frailty index, summary health risk (hierarchical condition category scores), county-level health-care environment, and zip code–level socioeconomic variables (Supplementary Methods A4).

We estimated the association of private equity acquisition and low-value treatment within each of the 5 subcohorts separately. We used a 2-way fixed-effects difference-in-differences model, accommodating staggered exposure initiation dates, with the following specification:


$$Y_{i,t(d)}{=\alpha +\lambda }_{t}{+\beta }_{1}{\left(\text{PE}\right)}_{i}{+\beta }_{2}{\left(\text{PE}\_\text{post}\right)}_{i,t(d)}{+\beta }_{3}{\left(X\right)}_{i,t(d)}{+\mu \text{TIN}}_{\left(i\right)}+\in_{i,t(d)}$$

where Y_i, t(d)_ is the outcome for patient *i* observed at their diagnosis date *t(d)*, λ indicates calendar year (*t*) fixed effects, *PE* an indicator (equal to 1 for patients whose physician was ever private equity acquired and 0 otherwise), *PE_post* an indicator for patients whose physician was private equity acquired as of their index diagnosis date, *X* refers to all covariates listed above, and *TIN* is tax identification number–level fixed effects for the tax identification number associated with patient *i*’s physician; β_2_ is the difference-in-differences estimate of interest. We conducted sensitivity analyses (1) adding radiation oncology as an eligible specialty type for oncologist assignment, (2) without tax identification number fixed effects, and (3) analyzing Medicare Advantage and traditional Medicare beneficiaries separately. We considered 2-sided *P *< .05 statistically significant.

The study comprised 141 223 patients contributing 201 348 observations (Figure S1, Table S3). The largest subcohort was biologics (*n* = 72 476 observations), and the smallest was denosumab (*n* = 9609). The proportion of private equity–exposed patients increased from 1.9% (584/31 236) in 2016 to 3.3% (1154/34 696) in 2020. Private equity–exposed (the treatment group) and private equity–unexposed patients (the control group) were largely similar (Tables S4-S8).

The unadjusted use of low-value treatment among unexposed patients was greatest for low-value biologics (79.3%) and lowest for addition of bevacizumab for ovarian cancer (7.5%) (Figure 1). Comparing patients treated by oncologists who had ever vs never practiced at a private equity–acquired address, the adjusted use of low-value treatment was not statistically different at baseline (the not–private equity acquired period for the treatment group): G-CSF, +1.1 percentage points (95% confidence interval [CI] = −1.2 to 3.4); denosumab, −5.5 percentage points (95% CI = −13.8 to 2.7); nab-paclitaxel, −0.0 percentage points (95% CI = −1.9 to 1.8); bevacizumab, +0.6 percentage points (95% CI = −4.2 to 5.3); and biologics, +2.6 percentage points (95% CI = −0.1 to 5.3) (Table 1).

**Figure 1.**
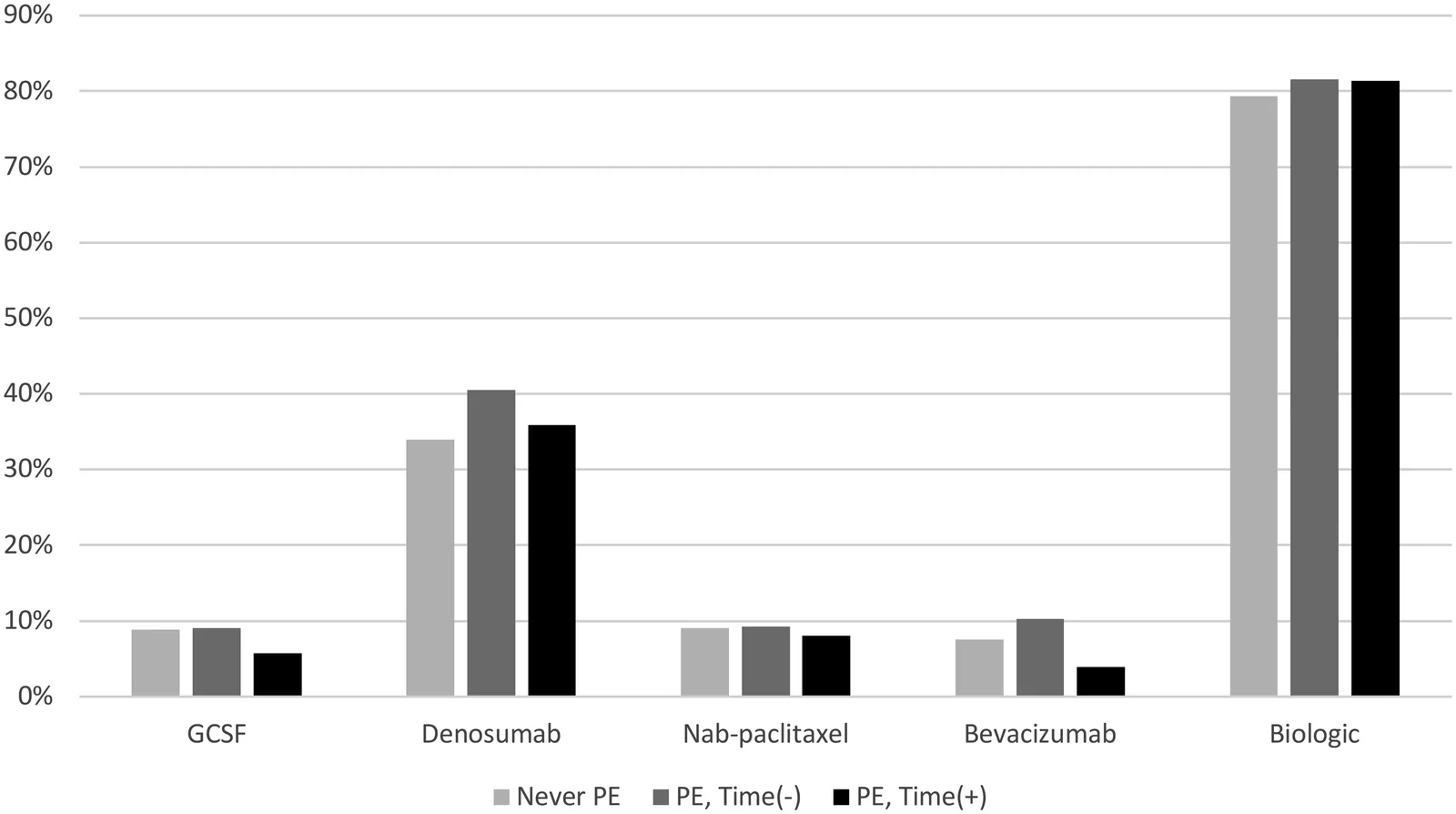
Unadjusted incidence of low-value cancer care, by exposure group. “Never private equity” (Never PE) indicates patients whose oncologist never practiced at a private equity–acquired address; “Private equity, Time(−)” indicates patients whose oncologist practiced at a private equity–acquired address but not on their diagnosis date; “Private equity, Time(+)” indicates patients whose oncologist practiced at a private equity–acquired address on their diagnosis date. G-CSF = granulocyte colony-stimulating factor.

**Table 1.** Adjusted prevalence of low-value cancer care.^a^

	Adjusted prevalence, % (95% CI)	Adjusted prevalence difference, percentage points (95% CI)	
Treatment	Control	Private equity at baseline vs control	Difference-in-differences estimate
**G-CSF**	8.8 (8.5 to 9.0)	1.1 (−1.2 to 3.4)	−2.8 (−5.5 to −0.2)
**Denosumab**	34.3 (33.4 to 35.1)	−5.5 (−13.8 to 2.7)	3.7 (−7.3 to 14.7)
**Nab-paclitaxel**	9.1 (8.9 to 9.3)	0.0 (−1.9 to 1.8)	−0.7 (−3.9 to 1.5)
**Bevacizumab**	7.6 (7.1 to 8.1)	0.6 (−4.2 to 5.3)	−6.5 (−12.5 to −0.4)
**Biologic**	79.4 (79.1 to 79.6)	2.6 (−0.1 to 5.3)	−2.9 (−6.0 to 0.2)

Abbreviations: CI = confidence interval; G-CSF = granulocyte colony-stimulating factor.

a “Control” indicates patients whose oncologist never practiced at a private equity–acquired address; “private equity” indicates patients whose oncologist ever practiced at a private equity–acquired address; “baseline” indicates patients whose oncologist practiced at a private equity–acquired address but not on their diagnosis date for the private equity group, and the overall period for the control group “difference-in-differences estimate” indicates the private equity acquisition effect estimate from the 2-way fixed-effects difference-in-differences model.

Comparing patients diagnosed when their oncologist was vs was not practicing at a private equity–acquired address, the unadjusted prevalence differences of low-value treatment were −3.3 percentage points for G-CSF, −4.6 percentage points for denosumab, −1.2 percentage points for nab-paclitaxel, −6.4 percentage points for bevacizumab, and −0.2 percentage points for biologics. After multivariable adjustment from the difference-in-differences estimation, the corresponding prevalence differences were −2.8 percentage points (95% CI = −5.5 to −0.2) for G-CSF, +3.7 percentage points (95% CI = −7.3 to 14.7) for denosumab, −0.7 percentage points (95% CI = −3.9 to 1.5) for nab-paclitaxel, −6.5 percentage points (95% CI = −12.5 to −0.4) for bevacizumab, and −2.9 percentage points (95% CI = −6.0 to 0.2) for biologics (Table 1). Results were similar when including radiation oncologists and removing tax identification number fixed effects (Tables S9 and S10). There were no consistent trends when analyzing Medicare Advantage and traditional Medicare beneficiaries separately (Table S11). Control and preintervention trends were not statistically different (difference-in-differences parallel trends assumption) (Figure S2).

Private equity ownership was not associated with increased prescribing of low-value cancer treatments. Private equity acquisition was not associated with differences in the use of 3 of the low-value treatments and was associated with reductions in 2 of the low-value treatments (G-CSF for low-risk chemotherapy and bevacizumab for ovarian cancer).

This result may be unexpected considering private equity firms’ incentives and strategy, which is to increase the profitability of acquired practices before “exit” or resale. The 5 low-value treatments we evaluated are all revenue-generating services, and their use (and overuse) would be aligned with the goal of increasing profitability. Studies of nononcology medical settings have found that private equity firms may use other mechanisms besides direct involvement in care delivery, such as consolidation, pricing, and staff reductions, to increase profitability.^3-8^ Further study is needed to assess whether these strategies are employed in oncology acquisitions, as well. For 2 low-value services (G-CSF and bevacizumab), use appeared unexpectedly lower in private equity–acquired firms, further supporting that increased drug utilization may not be a common firm strategy.

This finding differs from prior work suggesting that private equity acquisition affects care delivery: Private equity–acquired ophthalmology practices had both increased volume of intravitreal injections and increased cost per procedure due to substitution of more expensive drugs.^16^ The difference may be that services such as intravitreal injections are standardized, high-volume procedures and that substituting higher-priced drugs could substantially affect overall practice revenue, whereas the 5 low-value treatments we studied are relatively uncommon and therefore less likely to receive attention by practice ownership.

This study was limited to 5 low-value cancer treatments and did not analyze other services. Although we adjusted for patient-level and community-level factors, our results may have been biased by unmeasured confounders. Although we employed a difference-in-differences approach, comparing changes in care delivery by the same clinicians when practicing vs not practicing at private equity–acquired sites relative to changes in care by the clinicians who were never exposed to private equity, the finding may still be associative and may not imply causality. Due to small sample sizes, we were unable to conduct an event study analysis; this study therefore did not assess the possibility of heterogeneous treatment effects over time. Our results are limited to Medicare; future research should assess care delivery for commercially insured patients, for whom reimbursement is typically greater.^17^

## Supplementary Material

djag093_Supplementary_Data

## Data Availability

The data underlying this article were provided by the Centers for Medicare & Medicaid Services under license agreement, the terms of which preclude public sharing to maintain patient privacy. Data are available from the Centers for Medicare & Medicaid Services on request and establishment of data use agreement.
