# Supplementary material for: Use of low-value cancer care in practices acquired by private equity firms
Source: J Natl Cancer Inst. 2026 Mar 30;118(9):1758–61. doi: 10.1093/jnci/djag093 (PMC13421076; doi:10.1093/jnci/djag093)
Supplement: Supplementary file 1 [file djag093_supplementary_data.docx]

# **Supplementary Materials**

# **Supplementary Methods A1. Identifying the study population**

We identified Medicare beneficiaries, enrolled in either Traditional fee-for-service Medicare (TM) or a Medicare Advantage (MA) plan, who had a new diagnosis of cancer and a claim of cancer treatment by requiring them to meet at least one of the following criteria:

1) A claim in the Inpatient file containing a principal or other diagnosis of cancer.
2) A claim in the Outpatient file having both (1) a principal or other diagnosis of cancer and (2) a Healthcare Common Procedure Coding System (HCPCS) code mapping to the Restructured Berenson-Eggers Type of Services Classification System (RBCS) code associated with cancer treatment.
3) A claim in the Carrier file having both (1) a principal or other diagnosis of cancer and (2) a RBCS code associated with cancer treatment.
4) A claim in the Durable Medical Equipment (DME) file having both (1) a principal or other diagnosis of cancer and (2) a RBCS code associated with cancer treatment.
5) An event in the Part D event data file mapping to an NDC of a cancer drug.

*Defining index date and washout to exclude prevalent cases*: We included beneficiaries with an index date (defined as the date of first cancer diagnosis code) occurring in January 1 2016 – June 30, 2020. To avoid inclusion of beneficiaries with prevalent cancers, we included only index dates that met criteria 1-5 above, with 12 months of claims observed prior to the index date having no cancer claims.

*Assigning cancer site:* We assigned a patient’s cancer site based on a hierarchical algorithm validated against Surveillance, Epidemiology, and End Results (SEER) registry data (described next). We evaluated all cancer claims associated with treatments that occurred from the index date through 180 days afterward.

1) Beneficiaries with any inpatient facility claims were assigned the cancer site with the highest number of inpatient cancer claims (in cases of ties, to the cancer site with the highest paid amount of claims).
2) Beneficiaries with any hospital outpatient facility claims and no inpatient facility claims were assigned the cancer site with the highest number of hospital outpatient cancer claims (in cases of ties, to the cancer site with highest paid amount of claims).
3) Beneficiaries with Carrier file claims only were assigned the cancer site with the highest number of Carrier file cancer claims (in cases of ties, to the cancer site with the highest paid amount of claims).

*Coverage and other requirements*: We required all beneficiaries to have continuous enrollment in Medicare Parts A, B, and D, or Part C (Medicare Advantage) and D, from 365 days prior to their index date through the end of the cohort-specific outcome period (see below, Appendix A2). We also required beneficiaries to stay in one program (either MA or TM) during this period, not have end-stage renal disease, and reside in the 50 states or D.C.

# **Supplementary Methods** **A2. Cohort identification: Treatment-specific requirements**

*Cohort for the Analysis of Use of Granulocyte colony stimulating factor (GCSF)*

1) We identified beneficiaries with incident breast, lung, or colorectal cancer.
2) We required beneficiaries to have the first chemotherapy claim within 30 days before to 180 days after their index date.
3) We selected beneficiaries who were on a chemotherapy regimen with low (<10%) risk of neutropenic fever. Low-risk chemotherapy regimens we identified and compiled from the literature and guidelines, as in prior work.^1^
4) To identify the receipt of one of these regimens, we looked forward 30 days from the first chemotherapy claim. Beneficiaries were required to have received no other chemotherapy drugs beside the drug (or drugs) that comprised the regimen. In cases of multi-drug regimens, we also required that claims for each of the constituent drugs occur on the first day with any chemotherapy claims. Beneficiaries who were not categorized as having received low-risk chemotherapy, or who received multiple low-risk chemotherapy combinations, were excluded.

*Cohort for Denosumab Use among Patients with Castrate-Sensitive Prostate Cancer*

1) We identified beneficiaries with incident prostate cancer.
2) We excluded beneficiaries who had any claim with a diagnosis code for osteoporosis, osteopenia, hypercalcemia, or bone fracture prior to 180 days after the index date.
3) We excluded beneficiaries who had any claim for a bisphosphonate drug or denosumab more than 30 days prior to the index date.
4) We required beneficiaries to have bone metastasis, defined by one or more inpatient claims with any diagnosis code of bone metastasis, or two or more outpatient claims for treatments, diagnostic procedures, or evaluation and management with diagnosis codes for bone metastasis, occurring between 30 days prior to and 180 days after the index date.
5) We required beneficiaries to have a claim for androgen deprivation therapy (leuprolide, goserelin, triptorelin, abarelix, cetrorelix, ganirelix, degarelix, or surgical orchiectomy) or an antiandrogen drug (bicalutamide, flutamide, or nilutamide) occurring between 30 days prior to and 180 days after the index date.

*Cohort for Nab-paclitaxel Use (versus Paclitaxel)*

1) We identified beneficiaries with incident breast or lung cancer.
2) We included beneficiaries who had any claims for either paclitaxel or nab-paclitaxel within 365 days of the index date. Those with no claims for either drug within 365 days were excluded.

*Cohort for Ovarian Cancer, Use of Bevacizumab/Carboplatin/Paclitaxel (versus Carboplatin/Paclitaxel)*

1) We identified beneficiaries with incident ovarian cancer.
2) We included beneficiaries whose first chemotherapy regimen began within 365 days of the index date, and included the drugs carboplatin and paclitaxel initiating on the same day.

*Cohort for Biologic Use*

1) We included beneficiaries with incidence of any of the cancer types treated with one or more of the identified list of drugs with new biosimilar competition (Appendix Table A2).
2) We required beneficiaries to have their index date after the relevant biosimilar competitor entered the market (Appendix Table A2).
3) We required beneficiaries to fill either the biologic or biosimilar of interest within 365 days of the index date.

**Supplementary Methods A3. Private Equity Exposure**

First, each patient was assigned a primary oncologist based on the NPI appearing on the plurality of evaluation & management claims within 30 days before to 60 days after the index diagnosis date, following prior methods.^2^ We limited assignable physicians to those with a CMS specialty code related to oncology: 82 (hematology), 83 (hematology/oncology), 90 (medical oncology), 91 (surgical oncology), or 98 (gynecological oncology); in sensitivity analysis we also included 92 (radiation oncology).

Oncology practices (both medical and radiation) which were acquired by private equity (PE) firms during 2003-2022 were identified as previously described.^3^ The sources for oncology-related financial transactions were Capital IQ, CB Insights, Thomson ONE, and Pitchbook. The name of the acquired practice, acquiring PE firm, date of acquisition, and location of the acquired practice (state, city, and ZIP) were obtained from Pitchbook. Acquisitions were verified by comparison with other publicly available sources, including press releases.

Street addresses for each acquired practice location were then obtained via manual web search. We searched both current and archival (Wayback Machine) web data for institutional websites, map data, or financial transaction announcements which identified practices by street address.

Archival web data was searched in proximity to the date of acquisition. In cases where only one oncology practice was identified within the ZIP code obtained from Pitchbook, it was assumed to be the acquired practice. In cases where multiple oncology practices were found within a relevant ZIP, we continued to search for contextual information to identify which was the PE-acquired location. If a Pitchbook-identified acquisition could not confidently be paired with an oncology provider practice address, the PE acquisition was dropped from the dataset. Of our starting set of N=683 PE acquisitions, we were unable to identify the acquired practice and/or its street address in 50 cases, leaving 633 unique PE-acquired practice addressed for analysis.

We then mapped PE-acquired practice addresses to longitudinal National Plan & Provider Enumeration System data housed by the National Bureau of Economic Research, from 5/23/2005 to 7/12/2020.^4^ For each physician/NPI, NPPES data may contain multiple, historical practice addresses. We inferred the time period in which they practiced at each address by tracking their practice address across NPPES updates; when a new practice address appeared, we assumed that the physician began practicing at that address on the date of the first NPPES update which contained that address, and that 1 day prior was their final date of practice at the prior address. We were thereby able to identify the set of NPIs who, during 2005-2020, ever practiced at an address acquired by PE, and for what time interval they practiced there.

Exposure to PE was then defined on the patient level. We first identified patients whose assigned oncologist ever vs. never practiced (during 2005-2020) at an address that became PE-acquired at any point. Among those patients whose oncologist ever practiced at a PE-acquired address, we defined PE exposure (“Time(+)”) as both 1) oncologist was practicing at the PE-acquired address on the patient’s diagnosis date, and 2) diagnosis date occurred after the address’ acquisition date. Importantly, patients who where thereby defined as unexposed (“Time(-)”) included both those whose oncologists’ practices had not yet been acquired by PE and those whose oncologist had previously practiced at a PE-acquired location but had subsequently relocated.

# **Supplementary Methods A4. Explanatory variables**

Below we list explanatory variables and the datasets used to construct those covariates.

1) Demographics: age (age groups <65, 65-69,70-74,75-79,80-84,>=85), gender, race (non-Hispanic White, non-Hispanic Black, Hispanic, Asian, and other race), and Medicare-Medicaid dual eligibility. These variables were obtained from the Medicare Beneficiary Summary File (MBSF). To identify race, we used the RTI race code from MBSF.
2) Health risk measures: Cancer metastasis, claims-based frailty index, and a summary health risk score. To construct cancer metastatic status, we looked for ICD codes indicating metastasis (C77-C79) during the washout period including the trigger date. We required patients to have at least two claims of metastatic status in the OP or Carrier file. The claims-based frailty index was constructed following Kim et al’s. (2018) algorithm.^5^ This index accounts for functional limitations and differences in the propensity to receive chemotherapy treatments. The summary risk measure is the Hierarchical Condition Category (HCC) score used by CMS to adjust MA payments.^6,7^ We computed HCC scores using the v22 CMS-HCC software, excluding Health Risk Assessment (HRA)/chart review records for MA enrollees and HRA records for TM beneficiaries.
3) ZIP-level rurality of residence based on Rural-Urban Commuting Area codes.
4) County-level health care provider-related variables (hospital beds per 1000 residents, physicians per 1000, and skilled nursing facility beds per 1000) were obtained from the Area Health Resources File (AHRF),
5) Zip-level socioeconomic variables (percentage of adults aged 25 or older with a 4-year college degree, percentage of households living under the Federal Poverty Limit, and the percentage age 5 or older speaking English only) came from the American Community Survey (ACS).
6) Medicare Advantage enrollment (vs. traditional Medicare), obtained from CMS enrollment files.

**Table S1**. Low-value Cancer Treatments Examined in the Study. Abbreviations: NCCN: National Comprehensive Cancer Network; ASCO=American Society of Clinical Oncology; CALGB=Cancer and Leukemia Group B trial; STAMPEDE=Systemic Therapy in Advancing or Metastatic Prostate Cancer: Evaluation of Drug Efficacy; GOG: Gynecologic Oncology Group; ICON=International Collaboration on Ovarian Neoplasms; AURELIA=Avastin Use in Platinum-Resistant Epithelial Ovarian Cancer, NCT=National Clinical Trial; NA=Not applicable.

| Low-value Cancer Treatments | Guidelines or  Randomized Clinical Trials^*^ | Outcome specification |
| --- | --- | --- |
| *Non-recommended treatments* | |  |
| Granulocyte colony stimulating factors (GCSF) for primary prevention of neutropenic fever with chemotherapy that is low risk | NCCN guidelines,^8^ASCO Choosing Wisely,^9^ ASCO guidelines^10^ | Claim for any GCSF drug within 30 days after the first chemotherapy claim |
| Denosumab for skeletal-related event prevention in castration sensitive prostate cancer | CALGB 90202,^11^ STAMPEDE,^12^ NCCN guidelines,^13^expert opinion^14^ | Claim for denosumab within 30 days before to 180 days after index date |
| *High-cost treatments for which low-cost alternatives are available* | |  |
| Addition of bevacizumab to the combination of carboplatin and paclitaxel for ovarian cancer^§^ | GOG-0218,^15^ ICON-7,^16^ AURELIA,^17^ GOG-0213^18^ | Claim for bevacizumab on same day as carboplatin and paclitaxel |
| Nab-paclitaxel for breast or lung cancer | NCT00540514,^19^ CALGB 40502^20^ | First claim for a paclitaxel-based drug is nab-paclitaxel |
| Biologic drugs for which biosimilars are available  Bevacizumab for lung, colorectal, ovarian cancer  Trastuzumab for breast cancer  Rituximab for non-Hodgkin lymphoma  Peg-filgrastim for any cancer | NA^†^ | First claim for the index drug is for the originator biologic rather than a biosimilar version |

^*^ Basis for inclusion in the study, such as clinical trial evidence and clinical practice guidelines

^†^ Biosimilar drugs are considered therapeutically equivalent to biologics but have lower prices.

**Figure S1**. Identifying the study population. The study included for Medicare beneficiaries who had a new cancer diagnosis and a cancer treatment between 2016 and June 30, 2020, after having no cancer diagnosis or treatment claim during at least one prior year. Medicare Advantage (MA) enrollees were required to be in contracts that had highly complete encounter records. After all potentially eligible patients with the relevant cancer sites were identified, we then created 5 subcohorts (one for each low value treatment) comprising patients meeting the additional requirements detailed in **Supplementary Methods A2**. Each outcome was binary and observed only once for each patient, but patients could be included in multiple cohorts if they met the requirements of each; e.g., a patient with prostate cancer could be evaluated for both denosumab use (CSPC cohort) and peg-filgrastim use (Biosimilar cohort). MA, Medicare Advantage; TM, Traditional Medicare; ESRD, end stage renal disease; CML, chronic myeloid leukemia; GCSF, granulocyte colony stimulating factor; NHL, non-Hodgkin lymphoma.

# **Table S2.** Biosimilar availability dates

| Cancer types | Drug, biologic name | Drug, biosimilar name | First biosimilar availability date |
| --- | --- | --- | --- |
| Lung, colon, rectal | Avastin | bevacizumab-awwb (Mvasi), bevacizumab-bvzr (Zirabev) | 7/1/2019 |
| Breast | Herceptin | trastuzumab-anns (Kanjinti), trastuzumab-qyyp (Trazimera), trastuzumab-dttb (Ontruzant), trastuzumab-pkrb (Herzuma), trastuzumab-dkst (Ogivri) | 7/1/2019 |
| Non-Hodgkin lymphoma | Rituxan | rituximab-abbs (Truxima),  rituximab-pvvr (Ruxience),  rituximab-arrx (Riabni) | 11/1/2019 |
| Any cancer | Neulasta | pegfilgrastim-jmdb (Fulphila), pegfilgrastim-cbqv (Udenyca), pegfilgrastim-bmez (Ziextenzo),  pegfilgrastim-apgf (Nyvepria) | 6/4/2018 |

**Table S3**. Unadjusted sample characteristics. Numbers represent patient-subcohort observations. “Never PE” indicates patients whose oncologist never practiced at a PE-acquired address; “PE, Time(-)” indicates patients whose oncologist practiced at a PE-acquired address but not on their diagnosis date; “PE, Time(+)” indicates patients whose oncologist practiced at a PE-acquired address on their diagnosis date. GCSF, granulocyte colony stimulating factors; FPL, federal poverty limit; HCC, hierarchical condition category; SD, standard deviation; SNF, skilled nursing facility.

| Characteristics | Overall  (N = 201,348) | Never PE  (n = 192,338) | PE, Time(-)  (n = 3,764) | PE, Time(+)  (n = 5,246) |
| --- | --- | --- | --- | --- |
| Subcohort, No. (%) |  |  |  |  |
| GCSF | 45,100 | 42,898 | 1,016 | 1,186 |
| Denosumab | 9,609 | 9,249 | 190 | 170 |
| Nab-paclitaxel | 62,787 | 59,718 | 1,466 | 1,603 |
| Bevacizumab | 11,376 | 10,968 | 175 | 233 |
| Biologic | 72,476 | 69,505 | 917 | 2,064 |
| Index diagnosis year |  |  |  |  |
| 2016 | 31,236 (15.5%) | 29,772 (15.5%) | 880 (23.4%) | 584 (11.1%) |
| 2017 | 27,324 (13.6%) | 25,951 (13.5%) | 740 (19.7%) | 633 (12.1%) |
| 2018 | 42,988 (21.4%) | 41,109 (21.4%) | 946 (25.1%) | 933 (17.8%) |
| 2019 | 65,104 (32.3%) | 62,371 (32.4%) | 791 (21.0%) | 1,942 (37.0%) |
| 2020 | 34,696 (17.2%) | 33,135 (17.2%) | 407 (10.8%) | 1,154 (22.0%) |
| Gender, No. (%) |  |  |  |  |
| Female | 125,507 (62.3%) | 119,964 (62.4%) | 2,330 (61.9%) | 3,213 (61.2%) |
| Male | 75,841 (37.7%) | 72,374 (37.6%) | 1,434 (38.1%) | 2,033 (38.8%) |
| Age, No. (%) |  |  |  |  |
| Age < 65 | 19,135 (9.5%) | 18,209 (9.5%) | 363 (9.6%) | 563 (10.7%) |
| 65 ≤ Age < 70 | 48,431 (24.1%) | 56,536 (29.4%) | 868 (23.1%) | 1,293 (24.6%) |
| 70 ≤ Age < 75 | 59,312 (29.5%) | 41,947 (21.8%) | 1,138 (30.2%) | 1,638 (31.2%) |
| 75 ≤ Age < 80 | 43,807 (21.8%) | 20,562 (10.7%) | 799 (21.2%) | 1,061 (20.2%) |
| 80 ≤ Age < 85 | 21,473 (10.7%) | 8,814 (4.6%) | 427 (11.3%) | 484 (9.2%) |
| Age ≥ 85 | 9,190 (4.6%) | 19,973 (10.4%) | 169 (4.5%) | 207 (3.9%) |
| Race, No. (%) |  |  |  |  |
| Non-Hispanic White | 166,197 (82.5%) | 158,609 (82.5%) | 3,143 (83.5%) | 4,445 (84.7%) |
| Non-Hispanic Black | 20,900 (10.4%) | 19,973 (10.4%) | 416 (11.1%) | 511 (9.7%) |
| Hispanic | 8,807 (4.4%) | 8,480 (4.4%) | 140 (3.7%) | 187 (3.6%) |
| Asian | 3,619 (1.8%) | 3,531 (1.8%) | 32 (0.9%) | 56 (1.1%) |
| Other | 1,825 (0.9%) | 1,745 (0.9%) | 33 (0.9%) | 47 (0.9%) |
| Dual eligible, No. (%) | 39,759 (19.7%) | 37,951 (19.7%) | 745 (19.8%) | 1,063 (20.3%) |
| Medicare Advantage, No. (%) | 63,169 (31.4%) | 60,180 (31.3%) | 1,230 (32.7%) | 1,759 (33.5%) |
| HCC score, No. (%) |  |  |  |  |
| 0 ≤ HCC < 1 | 40,643 (20.2%) | 38,920 (20.2%) | 731 (19.4%) | 992 (18.9%) |
| 1 ≤ HCC < 2 | 53,434 (26.5%) | 51,109 (26.6%) | 946 (25.1%) | 1,379 (26.3%) |
| 2 ≤ HCC < 4 | 73,336 (36.4%) | 69,918 (36.4%) | 1,461 (38.8%) | 1,957 (37.3%) |
| HCC ≥ 4 | 33,935 (16.9%) | 32,391 (16.8%) | 626 (16.6%) | 918 (17.5%) |
| Frailty index, Mean (SD) | 0.179 (0.059) | 0.179 (0.059) | 0.182 (0.060) | 0.181 (0.059) |
| Rural residence, No. (%) | 52,140 (25.9%) | 49,380 (25.7%) | 1,010 (26.8%) | 1,750 (33.4%) |
| County-level characteristics, Mean (SD) |  |  |  |  |
| Inpatient beds/1000 population | 3.01 (2.27) | 3.01 (2.28) | 3.08 (1.91) | 2.99 (2.03) |
| Physicians/1000 population | 3.16 (2.35) | 3.18 (2.37) | 2.89 (1.99) | 2.61 (1.79) |
| Skilled Nursing Facility beds/1000 population | 5.97 (3.30) | 5.96 (3.30) | 6.04 (2.98) | 6.35 (3.72) |
| ZIP-level characteristics, Mean (SD) |  |  |  |  |
| % college educated | 29.6 (16.1) | 29.7 (16.2) | 26.4 (14.1) | 26.8 (14.6) |
| % speaking English only | 84.8 (16.3) | 84.7 (16.4) | 87.1 (14.1) | 88.9 (12.2) |
| % under FPL | 13.7 (8.3) | 13.6 (8.3) | 14.4 (8.4) | 14.4 (8.4) |

**Table S4**. Sample characteristics, GCSF subcohort. “Never PE” indicates patients whose oncologist never practiced at a PE-acquired address; “PE, Time(-)” indicates patients whose oncologist practiced at a PE-acquired address but not on their diagnosis date; “PE, Time(+)” indicates patients whose oncologist practiced at a PE-acquired address on their diagnosis date. Cells with fewer than 30 patients are masked to avoid re-identification. GCSF, granulocyte colony stimulating factors; FPL, federal poverty limit; HCC, hierarchical condition category; SD, standard deviation; SNF, skilled nursing facility.

| Characteristics | Never PE | PE, Time(-) | PE, Time(+) |
| --- | --- | --- | --- |
| (n=45,100) | (n=42,898) | (n=1,016) | (n=1,186) |
| Index diagnosis year, No. (%) |  |  |  |
| 2016 | 11,147 (26.0%) | 331 (32.6%) | 221 (18.6%) |
| 2017 | 8,968 (20.9%) | 271 (26.7%) | 226 (19.1%) |
| 2018 | 8,865 (20.7%) | 226 (22.2%) | 210 (17.7%) |
| 2019 | 9,677 (22.6%) | 135 (13.3%) | 368 (31.0%) |
| 2020 | 4,241 (9.9%) | 53 (5.2%) | 161 (13.6%) |
| Coverage Type, No. (%) |  |  |  |
| Traditional Medicare | 29,861 (69.6%) | 678 (66.7%) | 810 (68.3%) |
| Medicare Advantage | 13,037 (30.4%) | 338 (33.3%) | 376 (31.7%) |
| Gender, No. (%) |  |  |  |
| Female | 23,527 (54.8%) | 556 (54.7%) | 623 (52.5%) |
| Male | 19,371 (45.2%) | 460 (45.3%) | 563 (47.5%) |
| Age, No. (%) |  |  |  |
| Age < 65 | 4,205 (9.8%) | 104 (10.2%) | 124 (10.5%) |
| 65 ≤ Age < 70 | 12,463 (29.1%) | 222 (21.9%) | 257 (21.7%) |
| 70 ≤ Age < 75 | 10,082 (23.5%) | 299 (29.4%) | 379 (32.0%) |
| 75 ≤ Age < 80 | 5,112 (11.9%) | 234 (23.0%) | 248 (20.9%) |
| 80 ≤ Age < 85 | 1,854 (4.3%) | 112 (11.0%) | 124 (10.5%) |
| Age ≥ 85 | 4,578 (10.7%) | 45 (4.4%) | 54 (4.6%) |
| Race, No. (%) |  |  |  |
| Non-Hispanic White | 35,794 (83.4%) | 865 (85.1%) | 1,001 (84.4%) |
| Non-Hispanic Black | 4,578 (10.7%) | 103 (10.1%) | 126 (10.6%) |
| Hispanic | 1,524 (3.6%) | 30 (3.0%) | 37 (3.1%) |
| Asian | 629 (1.5%) | <30 | <30 |
| Other | 373 (0.9%) | <30 | <30 |
| Dual eligible, No. (%) | 9,776 (22.8%) | 219 (21.6%) | 279 (23.5%) |
| HCC score, No. (%) |  |  |  |
| 0 ≤ HCC < 1 | 6,450 (15.0%) | 143 (14.1%) | 147 (12.4%) |
| 1 ≤ HCC < 2 | 10,275 (24.0%) | 226 (22.2%) | 279 (23.5%) |
| 2 ≤ HCC < 4 | 17,857 (41.6%) | 452 (44.5%) | 532 (44.9%) |
| HCC ≥ 4 | 8,316 (19.4%) | 195 (19.2%) | 228 (19.2%) |
| Frailty index, Mean (SD) | 0.189 (0.060) | 0.190 (0.061) | 0.190 (0.060) |
| Metastatic diagnosis, No. (%) | 20,631 (48.1%) | 453 (44.6%) | 570 (48.1%) |
| Rural residence, No. (%) | 11,867 (27.7%) | 299 (29.4%) | 423 (35.7%) |
| Medical Care Access (County level), Mean (SD) |  |  |  |
| Inpatient beds/1000 population | 3.05 (2.40) | 3.02 (1.86) | 2.94 (1.91) |
| Physicians/1000 population | 3.04 (2.35) | 2.69 (1.95) | 2.46 (1.71) |
| Skilled Nursing Facility beds/1000 population | 6.11 (3.38) | 6.07 (2.92) | 6.48 (3.63) |
| Socio-economic variables (ZIP code level), Mean (SD) |  |  |  |
| % college educated | 27.8 (15.4) | 25.1 (13.6) | 25.1 (13.4) |
| % speaking English only | 85.5 (16.0) | 87.8 (13.8) | 88.7 (12.4) |
| % under federal poverty level | 14.4 (8.5) | 15.1 (8.4) | 15.2 (8.6) |

**Table S5**. Sample characteristics, denosumab subcohort. Cells with fewer than 30 patients are masked to avoid re-identification. FPL, federal poverty limit; HCC, hierarchical condition category; SD, standard deviation; SNF, skilled nursing facility.

| Characteristics | Never PE | PE, Time(-) | PE, Time(+) |
| --- | --- | --- | --- |
| (n=9,609) | (n=9,249) | (n=190) | (n=170) |
| Index diagnosis year, No. (%) |  |  |  |
| 2016 | 2,232 (24.1%) | 55 (28.9%) | 38 (22.4%) |
| 2017 | 1,958 (21.2%) | 45 (23.7%) | <30 |
| 2018 | 1,979 (21.4%) | 50 (26.3%) | <30 |
| 2019 | 2,065 (22.3%) | <30 | 43 (25.3%) |
| 2020 | 1,015 (11.0%) | <30 | <30 |
| Coverage Type, No. (%) |  |  |  |
| Traditional Medicare | 6,234 (67.4%) | 124 (65.3%) | 112 (65.9%) |
| Medicare Advantage | 3,015 (32.6%) | 66 (34.7%) | 58 (34.1%) |
| Gender, No. (%) |  |  |  |
| Female | 0 | 0 | 0 |
| Male | 9,249 (100.0%) | 190 (100.0%) | 170 (100.0%) |
| Age, No. (%) |  |  |  |
| Age < 65 | 303 (3.3%) | <30 | <30 |
| 65 ≤ Age < 70 | 2,120 (22.9%) | 33 (17.4%) | <30 |
| 70 ≤ Age < 75 | 2,069 (22.4%) | 44 (23.2%) | 48 (28.2%) |
| 75 ≤ Age < 80 | 1,663 (18.0%) | <30 | 34 (20.0%) |
| 80 ≤ Age < 85 | 1,521 (16.4%) | 36 (18.9%) | <30 |
| Age ≥ 85 | 1,201 (13.0%) | 37 (19.5%) | <30 |
| Race, No. (%) |  |  |  |
| Non-Hispanic White | 7,250 (78.4%) | 143 (75.3%) | 139 (81.8%) |
| Non-Hispanic Black | 1,201 (13.0%) | 31 (16.3%) | <30 |
| Hispanic | 529 (5.7%) | <30 | <30 |
| Asian | 163 (1.8%) | <30 | <30 |
| Other | 106 (1.1%) | <30 | <30 |
| Dual eligible, No. (%) | 1,546 (16.7%) | 33 (17.4%) | 31 (18.2%) |
| HCC score, No. (%) |  |  |  |
| 0 ≤ HCC < 1 | 2,036 (22.0%) | 41 (21.6%) | 41 (24.1%) |
| 1 ≤ HCC < 2 | 1,525 (16.5%) | 40 (21.1%) | <30 |
| 2 ≤ HCC < 4 | 3,325 (35.9%) | 64 (33.7%) | 63 (37.1%) |
| HCC ≥ 4 | 2,363 (25.5%) | 45 (23.7%) | <30 |
| Frailty index, Mean (SD) | 0.175 (0.062) | 0.173 (0.054) | 0.173 (0.064) |
| Metastatic diagnosis, No. (%) | 8,013 (86.6%) | 169 (88.9%) | 144 (84.7%) |
| Rural residence, No. (%) | 2,152 (23.3%) | 42 (22.1%) | 51 (30.0%) |
| Medical Care Access (County level), Mean (SD) |  |  |  |
| Inpatient beds/1000 population | 2.97 (2.12) | 3.59 (2.04) | 2.96 (2.25) |
| Physicians/1000 population | 3.41 (2.48) | 3.73 (2.52) | 2.67 (1.90) |
| Skilled Nursing Facilty beds/1000 population | 5.77 (3.20) | 6.09 (3.00) | 5.81 (3.55) |
| Socio-economic variables (ZIP code level), Mean (SD) |  |  |  |
| % college educated | 31.6 (17.0) | 29.0 (15.1) | 28.8 (15.7) |
| % speaking English only | 83.3 (17.3) | 85.6 (15.6) | 86.1 (15.0) |
| % under federal poverty level | 13.4 (8.4) | 14.1 (8.4) | 14.7 (9.4) |

**Table S6**. Sample characteristics, nab-paclitaxel subcohort. Cells with fewer than 30 patients are masked to avoid re-identification. FPL, federal poverty limit; HCC, hierarchical condition category; SD, standard deviation; SNF, skilled nursing facility.

| Characteristics | Never PE | PE, Time(-) | PE, Time(+) |
| --- | --- | --- | --- |
| (n=62,787) | (n=59,718) | (n=1,466) | (n=1,603) |
| Index diagnosis year, No. (%) |  |  |  |
| 2016 | 14,048 (23.5%) | 440 (30.0%) | 283 (17.7%) |
| 2017 | 12,469 (20.9%) | 393 (26.8%) | 321 (20.0%) |
| 2018 | 12,715 (21.3%) | 342 (23.3%) | 289 (18.0%) |
| 2019 | 14,192 (23.8%) | 196 (13.4%) | 467 (29.1%) |
| 2020 | 6,294 (10.5%) | 95 (6.5%) | 243 (15.2%) |
| Coverage Type, No. (%) |  |  |  |
| Traditional Medicare | 42,114 (70.5%) | 1,022 (69.7%) | 1,098 (68.5%) |
| Medicare Advantage | 17,604 (29.5%) | 444 (30.3%) | 505 (31.5%) |
| Gender, No. (%) |  |  |  |
| Female | 39,771 (66.6%) | 972 (66.3%) | 1,024 (63.9%) |
| Male | 19,947 (33.4%) | 494 (33.7%) | 579 (36.1%) |
| Age, No. (%) |  |  |  |
| Age < 65 | 6,872 (11.5%) | 172 (11.7%) | 199 (12.4%) |
| 65 ≤ Age < 70 | 17,538 (29.4%) | 344 (23.5%) | 415 (25.9%) |
| 70 ≤ Age < 75 | 12,539 (21.0%) | 440 (30.0%) | 506 (31.6%) |
| 75 ≤ Age < 80 | 5,757 (9.6%) | 317 (21.6%) | 295 (18.4%) |
| 80 ≤ Age < 85 | 1,932 (3.2%) | 149 (10.2%) | 138 (8.6%) |
| Age ≥ 85 | 6,943 (11.6%) | 44 (3.0%) | 50 (3.1%) |
| Race, No. (%) |  |  |  |
| Non-Hispanic White | 48,917 (81.9%) | 1,211 (82.6%) | 1,344 (83.8%) |
| Non-Hispanic Black | 6,943 (11.6%) | 184 (12.6%) | 182 (11.4%) |
| Hispanic | 2,362 (4.0%) | 47 (3.2%) | 49 (3.1%) |
| Asian | 961 (1.6%) | <30 | <30 |
| Other | 535 (0.9%) | <30 | <30 |
| Dual eligible, No. (%) | 13,351 (22.4%) | 321 (21.9%) | 374 (23.3%) |
| HCC score, No. (%) |  |  |  |
| 0 ≤ HCC < 1 | 14,395 (24.1%) | 327 (22.3%) | 338 (21.1%) |
| 1 ≤ HCC < 2 | 15,684 (26.3%) | 368 (25.1%) | 412 (25.7%) |
| 2 ≤ HCC < 4 | 20,635 (34.6%) | 545 (37.2%) | 606 (37.8%) |
| HCC ≥ 4 | 9,004 (15.1%) | 226 (15.4%) | 247 (15.4%) |
| Frailty index, Mean (SD) | 0.182 (0.059) | 0.185 (0.061) | 0.184 (0.059) |
| Metastatic diagnosis, No. (%) | 30,269 (50.7%) | 691 (47.1%) | 787 (49.1%) |
| Rural residence, No. (%) | 16,126 (27.0%) | 420 (28.6%) | 576 (35.9%) |
| Medical Care Access (County level), Mean (SD) |  |  |  |
| Inpatient beds/1000 population | 3.03 (2.37) | 3.04 (1.92) | 2.99 (1.96) |
| Physicians/1000 population | 3.06 (2.30) | 2.72 (1.94) | 2.51 (1.71) |
| Skilled Nursing Facility beds/1000 population | 6.03 (3.35) | 6.06 (2.84) | 6.45 (3.73) |
| Socio-economic variables (ZIP code level), Mean (SD) |  |  |  |
| % college educated | 28.3 (15.6) | 25.2 (13.8) | 25.7 (14.0) |
| % speaking English only | 85.1 (16.2) | 87.9 (13.7) | 88.9 (12.5) |
| % under federal poverty level | 14.3 (8.5) | 15.1 (8.7) | 15.1 (8.6) |

**Table S7**. Sample characteristics, bevacizumab subcohort. Cells with fewer than 30 patients are masked to avoid re-identification. FPL, federal poverty limit; HCC, hierarchical condition category; SD, standard deviation; SNF, skilled nursing facility.

| Characteristics | Never PE | PE, Time(-) | PE, Time(+) |
| --- | --- | --- | --- |
| (n=11,376) | (n=10,968) | (n=175) | (n=233) |
| Index diagnosis year, No. (%) |  |  |  |
| 2016 | 2,345 (21.4%) | 54 (30.9%) | 42 (18.0%) |
| 2017 | 2,360 (21.5%) | <30 | 45 (19.3%) |
| 2018 | 2,356 (21.5%) | 35 (20.0%) | 45 (19.3%) |
| 2019 | 2,633 (24.0%) | 34 (19.4%) | 65 (27.9%) |
| 2020 | 1,274 (11.6%) | <30 | 36 (15.5%) |
| Coverage Type, No. (%) |  |  |  |
| Traditional Medicare | 8,202 (74.8%) | 124 (70.9%) | 147 (63.1%) |
| Medicare Advantage | 2,766 (25.2%) | 51 (29.1%) | 86 (36.9%) |
| Gender, No. (%) |  |  |  |
| Female | 10,967 (100.0%) | 175 (100.0%) | 233 (100.0%) |
| Male | 0 | 0 | 0 |
| Age, No. (%) |  |  |  |
| Age < 65 | 693 (6.3%) | <30 | <30 |
| 65 ≤ Age < 70 | 3,221 (29.4%) | 42 (24.0%) | 64 (27.5%) |
| 70 ≤ Age < 75 | 2,562 (23.4%) | 59 (33.7%) | 63 (27.0%) |
| 75 ≤ Age < 80 | 1,299 (11.8%) | <30 | 51 (21.9%) |
| 80 ≤ Age < 85 | 556 (5.1%) | 32 (18.3%) | <30 |
| Age ≥ 85 | 736 (6.7%) | <30 | <30 |
| Race, No. (%) |  |  |  |
| Non-Hispanic White | 9,333 (85.1%) | 151 (86.3%) | 210 (90.1%) |
| Non-Hispanic Black | 736 (6.7%) | <30 | <30 |
| Hispanic | 585 (5.3%) | <30 | <30 |
| Asian | 223 (2.0%) | <30 | <30 |
| Other | 91 (0.8%) | <30 | <30 |
| Dual eligible, No. (%) | 1,642 (15.0%) | <30 | 31 (13.3%) |
| HCC score, No. (%) |  |  |  |
| 0 ≤ HCC < 1 | 716 (6.5%) | <30 | <30 |
| 1 ≤ HCC < 2 | 2,516 (22.9%) | 50 (28.6%) | <30 |
| 2 ≤ HCC < 4 | 5,467 (49.8%) | 73 (41.7%) | 118 (50.6%) |
| HCC ≥ 4 | 2,269 (20.7%) | <30 | 55 (23.6%) |
| Frailty index, Mean (SD) | 0.172 (0.055) | 0.171 (0.051) | 0.163 (0.050) |
| Metastatic diagnosis, No. (%) | 8,269 (75.4%) | 123 (70.3%) | 172 (73.8%) |
| Rural residence, No. (%) | 2,564 (23.4%) | 34 (19.4%) | 71 (30.5%) |
| Medical Care Access (County level), Mean (SD) |  |  |  |
| Inpatient beds/1000 population | 2.95 (2.06) | 2.93 (1.62) | 3.19 (2.11) |
| Physicians/1000 population | 3.33 (2.32) | 2.98 (1.61) | 2.73 (1.52) |
| Skilled Nursing Facility beds/1000 population | 5.76 (3.22) | 5.37 (2.91) | 7.17 (4.73) |
| Socio-economic variables (ZIP code level), Mean (SD) |  |  |  |
| % college educated | 32.5 (17.2) | 28.1 (13.7) | 27.0 (13.1) |
| % speaking English only | 83.3 (16.8) | 86.3 (13.4) | 88.6 (11.0) |
| % under federal poverty level | 12.8 (8.0) | 12.7 (7.6) | 13.4 (7.4) |

**Table S8**. Sample characteristics, biologic subcohort. Cells with fewer than 30 patients are masked to avoid re-identification. FPL, federal poverty limit; HCC, hierarchical condition category; SD, standard deviation; SNF, skilled nursing facility.

| Characteristics | Never PE | PE, Time(-) | PE, Time(+) |
| --- | --- | --- | --- |
| (n=72,476) | (n=69,505) | (n=917) | (n=2,054) |
| Index diagnosis year, No. (%) |  |  |  |
| 2016 | N/A | N/A | N/A |
| 2017 | N/A | N/A | N/A |
| 2018 | 15,194 (21.9%) | 293 (32.0%) | 354 (17.2%) |
| 2019 | 33,804 (48.6%) | 402 (43.8%) | 999 (48.6%) |
| 2020 | 20,311 (29.2%) | 221 (24.1%) | 695 (33.8%) |
| Coverage Type, No. (%) |  |  |  |
| Traditional Medicare | 45,747 (65.8%) | 586 (63.9%) | 1,320 (64.3%) |
| Medicare Advantage | 23,758 (34.2%) | 331 (36.1%) | 734 (35.7%) |
| Gender, No. (%) |  |  |  |
| Female | 45,699 (65.7%) | 627 (68.4%) | 1,333 (64.9%) |
| Male | 23,806 (34.3%) | 290 (31.6%) | 721 (35.1%) |
| Age, No. (%) |  |  |  |
| Age < 65 | 6,136 (8.8%) | 68 (7.4%) | 218 (10.6%) |
| 65 ≤ Age < 70 | 21,194 (30.5%) | 227 (24.8%) | 530 (25.8%) |
| 70 ≤ Age < 75 | 14,695 (21.1%) | 296 (32.3%) | 642 (31.3%) |
| 75 ≤ Age < 80 | 6,731 (9.7%) | 191 (20.8%) | 433 (21.1%) |
| 80 ≤ Age < 85 | 2,951 (4.2%) | 98 (10.7%) | 167 (8.1%) |
| Age ≥ 85 | 6,515 (9.4%) | 37 (4.0%) | 64 (3.1%) |
| Race, No. (%) |  |  |  |
| Non-Hispanic White | 57,315 (82.5%) | 773 (84.3%) | 1,751 (85.2%) |
| Non-Hispanic Black | 6,515 (9.4%) | 84 (9.2%) | 171 (8.3%) |
| Hispanic | 3,480 (5.0%) | 42 (4.6%) | 84 (4.1%) |
| Asian | 1,555 (2.2%) |  |  |
| Other | 640 (0.9%) |  |  |
| Dual eligible, No. (%) | 11,636 (16.7%) | 144 (15.7%) | 348 (16.9%) |
| HCC score, No. (%) |  |  |  |
| 0 ≤ HCC < 1 | 15,323 (22.0%) | 206 (22.5%) | 452 (22.0%) |
| 1 ≤ HCC < 2 | 21,109 (30.4%) | 262 (28.6%) | 616 (30.0%) |
| 2 ≤ HCC < 4 | 22,634 (32.6%) | 327 (35.7%) | 638 (31.1%) |
| HCC ≥ 4 | 10,439 (15.0%) | 122 (13.3%) | 348 (16.9%) |
| Frailty index, Mean (SD) | 0.173 (0.057) | 0.172 (0.057) | 0.175 (0.058) |
| Metastatic diagnosis, No. (%) | 30,608 (44.0%) | 411 (44.8%) | 911 (44.4%) |
| Rural residence, No. (%) | 16,671 (24.0%) | 215 (23.4%) | 629 (30.6%) |
| Medical Care Access (County level), Mean (SD) |  |  |  |
| Inpatient beds/1000 population | 2.98 (2.18) | 3.13 (1.97) | 3.00 (2.11) |
| Physicians/1000 population | 3.30 (2.41) | 3.19 (1.99) | 2.76 (1.91) |
| Skilled Nursing Facility beds/1000 population | 5.87 (3.21) | 6.10 (3.27) | 6.15 (3.63) |
| Socio-economic variables (ZIP code level), Mean (SD) |  |  |  |
| % college educated | 31.4 (16.7) | 29.0 (14.6) | 28.4 (15.6) |
| % speaking English only | 84.1 (16.5) | 85.4 (14.6) | 89.3 (11.5) |
| % under federal poverty level | 12.7 (8.0) | 12.9 (7.7) | 13.3 (8.1) |

# **Table S9.** Sensitivity analysis including radiation oncology as an assignable specialty type. “Control” indicates patients whose oncologist never practiced at a PE-acquired address; “PE” indicates patients whose oncologist ever practiced at a PE-acquired address; “DID estimate” indicates the PE acquisition effect estimate from the two-way fixed effects difference-in-differences model.

|  | Adjusted Prevalence, % (95% CI) | Adjusted Prevalence Difference, %-points  (95% CI) | |
| --- | --- | --- | --- |
|  | Control | PE vs. Control | DID estimate |
| GCSF | 8.7  (8.5 to 9.0) | 0.0  (-1.6 to 1.7) | -2.8  (-5.1 to -0.6) |
| Denosumab | 32.8  (31.9 to 33.7) | 4.7  (-1.1 to 10.5) | -7.4  (-15.5 to 0.6) |
| Nab-paclitaxel | 9.0  (8.8 to 9.2) | 0.0  (-1.4 to 1.4) | -1.3  (-3.2 to 0.6) |
| Bevacizumab | 7.5  (7.0 to 8.0) | 3.3  (-0.6 to 7.2) | -7.1  (-12.2 to -2.0) |
| Biologic | 79.4  (79.1 to 79.6) | -1.6  (-2.4 to 2.6) | 3.5  (0.5 to 6.5) |

# **Table S10.** Sensitivity analysis without TIN-level fixed effects. “Control” indicates patients whose oncologist never practiced at a PE-acquired address; “PE” indicates patients whose oncologist ever practiced at a PE-acquired address; “DID estimate” indicates the PE acquisition effect estimate from the two-way fixed effects difference-in-differences model.

|  | Adjusted Prevalence, % (95% CI) | Adjusted Prevalence Difference, %-points  (95% CI) | |
| --- | --- | --- | --- |
|  | Control | PE at baseline vs. Control | DID estimate |
| GCSF | 8.8  (8.6 to 9.1) | 0.3  (-1.5 to 2.1) | -3.2  (-5.5 to -0.8) |
| Denosumab | 34.0  (33.1 to 35.0) | 5.2  (-1.5 to 11.9) | -4.5  (-14.2 to 5.1) |
| Nab-paclitaxel | 9.1  (8.9 to 9.3) | 0.3  (-1.1 to 1.8) | -1.2  (-3.2 to 0.8) |
| Bevacizumab | 7.5  (7.0 to 8.0) | 3.3  (-0.6 to 7.1) | -7.0  (-12.1 to -2.0) |
| Biologic | 79.3  (79.0 to 79.6) | -0.4  (-2.1 to 3.0) | 2.8  (-0.3 to 5.9) |

**Table S11**: Subgroup analysis by patient insurance type, traditional Medicare and Medicare Advantage. “Control” indicates patients whose oncologist never practiced at a PE-acquired address; “PE” indicates patients whose oncologist ever practiced at a PE-acquired address; “DID estimate” indicates the PE acquisition effect estimate from the two-way fixed effects difference-in-differences model.

|  | Adjusted Prevalence, % (95% CI) | Adjusted Prevalence Difference, % (95% CI) | |
| --- | --- | --- | --- |
|  |  |  |  |
|  | Control | PE vs. Control | DID estimate |
| Traditional Medicare | | | |
| GCSF | 9.4 | 0.1 | -3.7 |
|  | (9.1 to 9.8) | (-2.1 to 2.3) | (-6.7 to -0.7) |
| CSPC | 36.7 | 3.9 | 1.8 |
|  | (35.5 to 37.9) | (-4.6 to 12.4) | (-10.4 to 14.0) |
| Paclitaxel | 9.5 | 0.7 | -1.7 |
|  | (9.2 to 9.8) | (-1.1 to 2.5) | (-4.2 to 0.8) |
| Ovarian | 7.2 | 3.6 | -7.2 |
|  | (6.7 to 7.8) | (-1.0 to 8.1) | (-13.3 to -1.1) |
| Biosimilar | 81.1 | 1.7 | 0.7 |
|  | (80.8 to 81.5) | (-1.4 to 4.8) | (-3.0 to 4.4) |
|  |  |  |  |
| Medicare Advantage | | | |
| GCSF | 7.4 | 0.7 | -1.8 |
|  | (7.0 to 7.9) | (-2.1 to 3.5) | (-5.7 to 2.0) |
| CSPC | 28.5 | 7.2 | -17.3 |
|  | (26.9 to 30.1) | (-3.7 to 18.2) | (-33.1 to -1.5) |
| Paclitaxel | 8.1 | -0.5 | -0.1 |
|  | (7.7 to 8.5) | (-3.0 to 2.1) | (-3.6 to 3.3) |
| Ovarian | 8.3 | 2.6 | -7.0 |
|  | (7.3 to 9.3) | (-5.0 to 10.1) | (-16.5 to 2.4) |
| Biosimilar | 75.8 | -1.7 | 6.4 |
|  | (75.3 to 76.4) | (-6.2 to 2.8) | (1.0 to 11.8) |

**Figure S2**: Trends in low-value care among control and pre-acquisition PE groups. Each panel shows the proportion of patients who received the corresponding low-value service in each calendar year, comparing the control group (patients treated in practices never acquired by PE) to the pre-intervention group (patients treated in practices that had not yet been acquired by PE but were acquired later in the study period). Differences in trends were assessed by applying an F-test to assess the joint significance of the coefficients on the interaction terms between the PE group indicator and calendar year dummies.

GCSF

|  | Control | Pre-acquisition |
| --- | --- | --- |
| 2016 | 0.0972459 | 0.132931 |
| 2017 | 0.0902096 | 0.0701107 |
| 2018 | 0.0869712 | 0.0752212 |
| 2019 | 0.0820502 | 0.0592593 |
| 2020 | 0.0789908 | 0.0754717 |

p = 0.11

Denosumab

|  | Control | Pre-acquisition |
| --- | --- | --- |
| 2016 | 0.369176 | 0.454545 |
| 2017 | 0.368744 | 0.4 |
| 2018 | 0.343103 | 0.48 |
| 2019 | 0.303148 | 0.25 |
| 2020 | 0.285714 | 0.25 |

p = 0.46

Nab-paclitaxel

|  | Control | Pre-acquisition |
| --- | --- | --- |
| 2016 | 0.111617 | 0.113636 |
| 2017 | 0.0826851 | 0.101781 |
| 2018 | 0.0789619 | 0.0847953 |
| 2019 | 0.0903326 | 0.0510204 |
| 2020 | 0.0865904 | 0.0736842 |

p = 0.23

Bevacizumab

|  | Control | Pre-acquisition |
| --- | --- | --- |
| 2016 | 0.0332623 | 0.111111 |
| 2017 | 0.0271186 | 0.0333333 |
| 2018 | 0.0831919 | 0.0857143 |
| 2019 | 0.115837 | 0.0882353 |
| 2020 | 0.142072 | 0.227273 |

p = 0.29

Biologic

|  | Control | Pre-acquisition |
| --- | --- | --- |
| 2017 | 0.97449 | 1 |
| 2018 | 0.944386 | 0.955631 |
| 2019 | 0.78399 | 0.78607 |
| 2020 | 0.693565 | 0.683258 |

p = 0.95

**References**

1.	Mitchell AP, Dusetzina SB, Mishra Meza A, Trivedi NU, Bach PB, Winn AN. Pharmaceutical industry payments and delivery of non-recommended and low value cancer drugs: population based cohort study. *BMJ*. 2023;2023(383:e075512). doi:10.1136/bmj-2023-075512

2.	Fishman E, Barron J, Liu Y, et al. Using claims data to attribute patients with breast, lung, or colorectal cancer to prescribing oncologists. *Pragmat Obs Res*. 2019;10:15-22. doi:10.2147/POR.S197252

3.	Tyan K, Lam MB, Milligan M. Private Equity Acquisition of Oncology Clinics in the US From 2003 to 2022. *JAMA Intern Med*. 2023;183(6):621-623. doi:10.1001/jamainternmed.2023.0334

4.	Roth J. National Plan and Provider Enumeration System (NPPES). Published online June 1, 2024. Accessed September 1, 2024. https://www.nber.org/research/data/national-plan-and-provider-enumeration-system-nppes

5.	Kim DH, Glynn RJ, Avorn J, et al. Validation of a Claims-Based Frailty Index Against Physical Performance and Adverse Health Outcomes in the Health and Retirement Study. *J Gerontol A Biol Sci Med Sci*. 2019;74(8):1271-1276. doi:10.1093/gerona/gly197

6.	Pope GC, Kautter J, Ellis RP, et al. Risk adjustment of Medicare capitation payments using the CMS-HCC model. *Health Care Financ Rev*. 2004;25(4):119-141.

7.	Medicare Risk Adjustment. Published online October 24, 2024. Accessed April 29, 2025. https://www.cms.gov/medicare/payment/medicare-advantage-rates-statistics/risk-adjustment

8.	Myeloid Growth Factors: NCCN Clinical Practice Guidelines in Oncology. Published online August 3, 2018.

9.	Five More Things Physicians and Patients Should Question. Published online October 29, 2013. Accessed November 25, 2017. http://www.choosingwisely.org/wp-content/uploads/2015/02/ASCO-Choosing-Wisely-List.pdf

10.	Smith TJ, Bohlke K, Lyman GH, et al. Recommendations for the Use of WBC Growth Factors: American Society of Clinical Oncology Clinical Practice Guideline Update. *J Clin Oncol*. 2015;33(28):3199-3212. doi:10.1200/JCO.2015.62.3488

11.	Smith MR, Halabi S, Ryan CJ, et al. Randomized controlled trial of early zoledronic acid in men with castration-sensitive prostate cancer and bone metastases: results of CALGB 90202 (alliance). *J Clin Oncol*. 2014;32(11):1143-1150. doi:10.1200/JCO.2013.51.6500

12.	James ND, Sydes MR, Clarke NW, et al. Addition of docetaxel, zoledronic acid, or both to first-line long-term hormone therapy in prostate cancer (STAMPEDE): survival results from an adaptive, multiarm, multistage, platform randomised controlled trial. *Lancet*. 2016;387(10024):1163-1177. doi:10.1016/S0140-6736(15)01037-5

13.	NCCN Clinical Practice Guidelines in Oncology: Prostate Cancer, Version 4.2019. Published online August 19, 2019. Accessed January 17, 2020. https://www.nccn.org/professionals/physician_gls/pdf/prostate.pdf

14.	Gillessen S, Attard G, Beer TM, et al. Management of Patients with Advanced Prostate Cancer: The Report of the Advanced Prostate Cancer Consensus Conference APCCC 2017. *Eur Urol*. 2018;73(2):178-211. doi:10.1016/j.eururo.2017.06.002

15.	Tewari KS, Burger RA, Enserro D, et al. Final Overall Survival of a Randomized Trial of Bevacizumab for Primary Treatment of Ovarian Cancer. *J Clin Oncol*. 2019;37(26):2317-2328. doi:10.1200/JCO.19.01009

16.	Oza AM, Cook AD, Pfisterer J, et al. Standard chemotherapy with or without bevacizumab for women with newly diagnosed ovarian cancer (ICON7): overall survival results of a phase 3 randomised trial. *Lancet Oncol*. 2015;16(8):928-936. doi:10.1016/S1470-2045(15)00086-8

17.	Pujade-Lauraine E, Hilpert F, Weber B, et al. Bevacizumab combined with chemotherapy for platinum-resistant recurrent ovarian cancer: The AURELIA open-label randomized phase III trial. *J Clin Oncol*. 2014;32(13):1302-1308. doi:10.1200/JCO.2013.51.4489

18.	Coleman RL, Brady MF, Herzog TJ, et al. Bevacizumab and paclitaxel-carboplatin chemotherapy and secondary cytoreduction in recurrent, platinum-sensitive ovarian cancer (NRG Oncology/Gynecologic Oncology Group study GOG-0213): a multicentre, open-label, randomised, phase 3 trial. *Lancet Oncol*. 2017;18(6):779-791. doi:10.1016/S1470-2045(17)30279-6

19.	Socinski MA, Bondarenko I, Karaseva NA, et al. Weekly nab-paclitaxel in combination with carboplatin versus solvent-based paclitaxel plus carboplatin as first-line therapy in patients with advanced non-small-cell lung cancer: final results of a phase III trial. *J Clin Oncol*. 2012;30(17):2055-2062. doi:10.1200/JCO.2011.39.5848

20.	Rugo HS, Barry WT, Moreno-Aspitia A, et al. Randomized Phase III Trial of Paclitaxel Once Per Week Compared With Nanoparticle Albumin-Bound Nab-Paclitaxel Once Per Week or Ixabepilone With Bevacizumab As First-Line Chemotherapy for Locally Recurrent or Metastatic Breast Cancer: CALGB 40502/NCCTG N063H (Alliance). *J Clin Oncol*. 2015;33(21):2361-2369. doi:10.1200/JCO.2014.59.5298
